# Life Expectancy Trends Among Integrated Health Care System Enrollees, 2014–2017

**DOI:** 10.7812/TPP/20.286

**Published:** 2021-12-14

**Authors:** Tori Finch, M Cabell Jonas, Kevin Rubenstein, Eric Watson, Sundeep Basra, Jose Martinez, Michael Horberg

**Affiliations:** ^1^ Mid-Atlantic Permanente Medical Group, Rockville, MD; ^2^ Mid-Atlantic Permanente Research Institute, Rockville, MD

**Keywords:** integrated care delivery, life expectancy, life tables, race- and ethnicity-specific life expectancy, sex-specific life expectancy

## Abstract

**Introduction:**

The Centers for Disease Control and Prevention (CDC) has reported downward trends in life expectancy and racial/ethnic differences between 2014 and 2017.

**Objective:**

To determine the life expectancy of the Kaiser Permanente Mid-Atlantic States (KPMAS) insured population as compared to the CDC National Vital Statistics data from 2014 to 2017. We also aimed to highlight the utilization of membership data to inform population statistical estimates such as life expectancy. We examine whether national trends in life expectancy are reflected in an insured population with relatively uniform access to care.

**Methods:**

This retrospective, data only study examined life expectancy between 2014 and 2017. Data from electronic medical records and the National Death Index were combined to construct complete life tables by race and sex for the KPMAS population, which was compared to the CDC National Vital Statistics data.

**Results:**

From 2014 to 2017, the overall KPMAS population life expectancy at birth varied between 84.6 and 85.2 years compared to the CDC reported national average of 78.6-78.9 years (p < 0.001). While the CDC dataset reported a 3.5- to 3.7-year life expectancy gap between non-Hispanic White and non-Hispanic Black populations, in the KPMAS population, this gap was significantly smaller (0.0-0.9 years). The gap in life expectancy between males and females was consistent across KPMAS and the CDC data; however, overall KPMAS male and female patient life expectancy was extended in comparison.

**Conclusion:**

Among members who disclosed their race/ethnicity, KPMAS Hispanic, non-Hispanic Black, and non-Hispanic White members had significantly higher life expectancies than the CDC dataset in all years reported.

## INTRODUCTION

Life expectancy has long been used as a population-based indicator of health as it reflects improvements and changes in public health, health care, economic conditions, and social factors.^1^^,^^2^ Since 2014, the Centers for Disease Control and Prevention’s (CDC’s) National Center for Health Statistics has reported a decline in US life expectancy.^3^^,^^4^ The reasons for this decline are varied and include factors that impact uninsured and insured populations alike, including drug overdose (particularly related to the opioid crisis), suicide, and disease (liver disease, metabolic diseases).^5^^–^^8^

Life expectancy disparities by sex, race/ethnicity, and other factors have been widely reported in the United States.^8^^–^^11^ However, research into the precise mechanisms driving these differences is ongoing. Complex variations in lifestyle, environment, education, social factors, economic variables, health status, and care access, as well as biological or genetic factors and more, exert influence.^8^^,^^12^^–^^16^ Even globally, socioeconomic status and comorbidities have a significant impact on life expectancy.^17^^–^^19^ Studies have indicated that being uninsured or underinsured can also influence mortality and life expectancy.^20^^–^^23^ One difficulty in exploring the relationship between insurance and life expectancy is that public datasets, such as the CDC National Vital Statistics data, do not include details regarding insurance status and line of business (public, private).

When faced with disparities and declines in national life expectancy, health care delivery systems have an opportunity to examine the health of insured populations served to understand whether national trends are reflected locally and to understand trends in insured populations. Delivery systems can then explore potential health care or social interventions to offset any disparities present.^24^^–^^27^ More broadly, understanding the life expectancy trends within an insured population contributes meaningful information to the conversation around the value of expanding health insurance to reach all populations.

Kaiser Permanente Mid-Atlantic States (KPMAS) is an integrated care delivery system comprised of a partnership between the Kaiser Foundation Health Plan and the Mid-Atlantic Permanente Medical group, which serves over 760,000 members in Washington DC, Virginia, and Maryland.^28^ The organization offers comprehensive preventive and acute care at over 48 clinical sites in the region. All members of the KPMAS system have health insurance and comprehensive access to care with the same medical teams available.

## OBJECTIVE

In response to the declines in life expectancy seen in the CDC’s National Center for Health Statistics dataset, KPMAS sought to examine life expectancy trends within the organization’s insured population across the same period, to determine if national trends were also reflected locally. Because of the broad uniformity in care across communities and time, and the presence of health insurance, KPMAS was in a strong position to examine whether national trends, including declining life expectancy and differences by race/ethnicity, were reflected in its member population.

## METHODS

### Design

To estimate life expectancy of KPMAS members, we utilized existing information from members’ electronic health records. Members were included in the study if they had active coverage on July 1st of any year from 2014 to 2017, or if they had died any time between 2014 and 2017 with active membership in a KPMAS health plan. Electronic health record information includes sex, self-reported race/ethnicity, and date of birth. Death records were retrieved from the National Death Index and matched to KPMAS records.^29^

### Main Outcomes

#### Race/Ethnicity

The race/ethnicity groupings utilized in the study were self-reported by members and included: Asian and Pacific Islander, Hispanic, non-Hispanic Black, non-Hispanic White, and other; the data for additional race/ethnicity categories was too limited to include. The CDC does not report life expectancy for Asian and Pacific Islanders, so no race/ethnicity equivalent comparison group was available. For the analysis of life expectancy by racial background, a “nonreporter” category was used for members who chose not to disclose their racial background. Additional analyses for nonreporters were conducted using the Geographically Enriched Member Sociodemographics database that imputes the probability that a member would identify as belonging to each race/ethnicity category.^30^ Race/ethnicity probabilities are computed by the Bayesian Improved Surname Geocoding method, which utilizes member’s surname, home address, and US census information (Supplementary Material).^31^^,^^32^

#### Life Expectancy Calculations

A modified version of the process described by Anderson^33^ was employed to construct complete life tables for the KPMAS population for the years 2014-2017. Modifications to the Anderson Mortality Rate Algorithm included not utilizing age misspecification corrections, as our birth date data is confirmed when members access medical care. Observations of members over the age of 100 were aggregated to produce a single mortality rate, which was applied to all ages up to 120. This methodology produced a death rate table, which was used to compute the life expectancy of the associated population using the Anderson Life Expectancy Algorithm.

#### Confidence Interval Calculation

Confidence intervals were calculated on all death rates and life expectancies using a parametric bootstrapping methodology. For each subpopulation, we constructed the associated death rate table, then, for each age *a*, we randomly sampled a new observation from a binomial distribution with *N* as the number of members having age *a* and p as the empirical death rate of members having age *a*.

This simulation produced a new death rate for each age, which was used to construct a new Life Expectancy table. One thousand bootstrap simulations were performed for each subpopulation; the 2.5th and 97.5th percentiles of these observations were taken as the bounds for the bootstrap 95% confidence interval.

#### p Value Calculation

Unless otherwise noted, statistical significance tests were computed using bootstrapping resampling techniques, as described by Efron.^34^ We generated bootstrap samples assuming the null hypothesis and examined the frequency with which the simulated data was as extreme as, or more extreme than, the observed data.

To perform this resampling, we assume the null value’s death rate by age is accurate. For comparisons against the CDC data, this is the national average; for comparisons between two KPMAS populations by sex or race/ethnicity, this is the average of their respective values, weighted by sample size. We then compute the life expectancy from the resampled death rate tables. p values are computed by comparing the observed life expectancy to the distribution of life expectancies estimated under the assumed null value’s death rate using 1,000 bootstrap iterations.^35^

#### Excess Life Graphs

**Figure 1.**
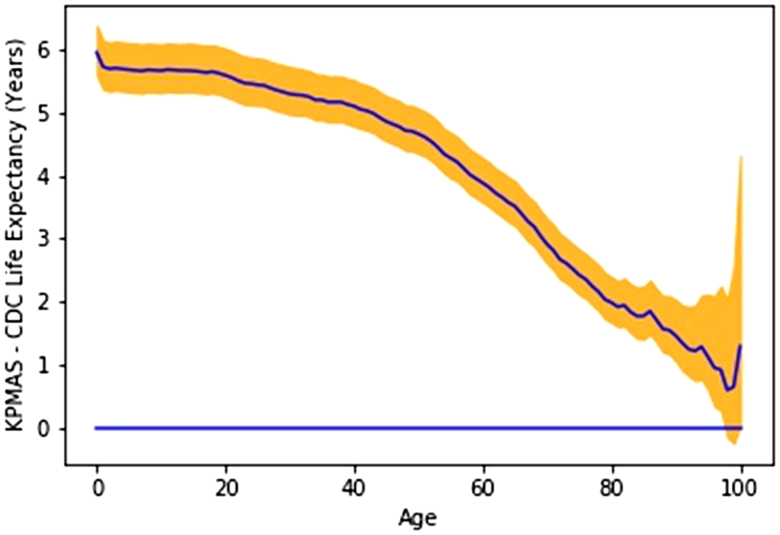
Overall excess life expectancy for all Kaiser Permanente Mid-Atlantic States members over centers for Disease Control and Prevention dataset, with 95% confidence intervals shown in colored shading (2015). CDC = Centers for Disease Control and Prevention; KPMAS = Kaiser Permanente Mid-Atlantic States.

**Figure 2.**
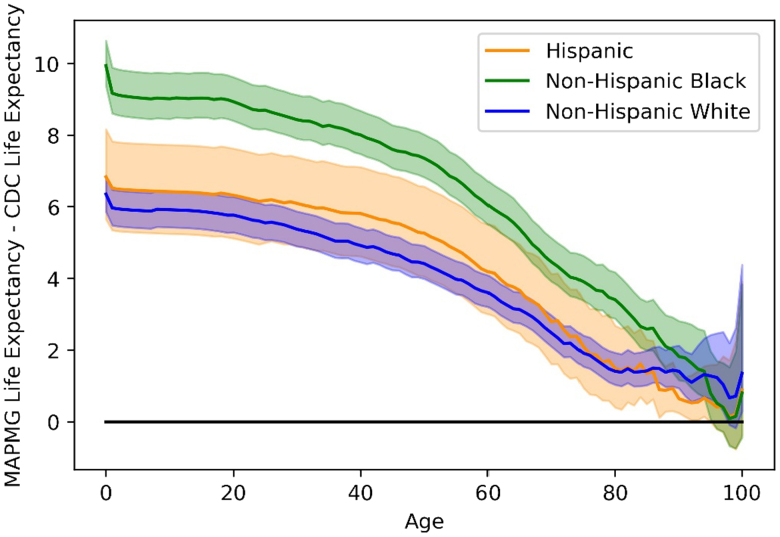
Overall excess life expectancy for all Kaiser Permanente Mid-Atlantic States Members Over Centers for Disease Control and Prevention dataset, by race, with 95% confidence intervals shown in colored shading (2015). CDC = Centers for Disease Control and Prevention; MAPMG = Mid-Atlantic Permanente Medical Group.

The Excess Life Expectancy plots compare the life expectations of 2 populations, the KPMAS population and the CDC dataset (Figures 1 and 2). Excess Life Expectancy plots for the year 2015 are shown, as 2015 represents one of the central years in the 2014-2017 study period; year-over-year differences for other years were minimal and did not change study results or conclusions (data not shown). At each point in the plot, the line represents the difference between the life expectancy of the first population and the second. Some plots also included confidence intervals computed using the bootstrapping method described above.

## RESULTS

### Demographics

During the study period, the KPMAS membership population grew from approximately 513,000 to approximately 697,000 members. Table 1 shows summary statistics on the characteristics of this population.[ID]TBL1[/ID] Across all 4 years, KPMAS had slightly more female than male members. Members who did not identify as either female or male were excluded from sex-based breakdowns. The KPMAS population is highly diverse across racial backgrounds with the largest group being non-Hispanic Black (36%-38%) and non-Hispanic White (27%-31%; Table 1). Additionally, between 9% and 10% of members do not report their race or ethnicity in each year of the study period. Table 1 also shows a breakdown of members by insurance type, including the categories Medicare, Medicaid, and Commercial (includes Exchanges). During the study period, between 73% and 77% of members were retained within the KPMAS-insured population from year to year. Retention was not measured against the KPMAS population predating the study period. In addition, retention is only presented with respect to the previous year, not with respect to any prior years.

**Table 1.** Kaiser Permanente Mid-Atlantic States membership demographic summary

	2014	2015	2016	2017
Sex distribution				
Male	47.5%	47.6%	47.4%	47.3%
Female	52.5%	52.4%	52.6%	52.7%
Race/ethnicity distribution				
Asian and Pacific Islanders	9.9%	10.9%	11.7%	12.2%
Hispanic	9.8%	11.3%	11.8%	12.0%
Non-Hispanic Black	38.1%	36.3%	36.6%	36.9%
Non-Hispanic White	31.0%	28.9%	27.9%	27.2%
Other	2.2%	2.3%	2.4%	2.6%
Nonreporter	9.0%	10.2%	9.5%	9.2%
Insurance funding				
Commercial	87.6%	84.2%	81.0%	79.0%
Medicaid	1.2%	5.5%	8.7%	10.7%
Medicare	11.2%	10.3%	10.3%	10.4%
Member retention				
(Year-over-year)		72.8%	76.5%	77.4%

### Life Expectancy

From 2014 to 2017 the Overall Life Expectancy for members of KPMAS was significantly higher than the national averages reported by the CDC (Table 2). The overall KPMAS population life expectancy at birth was between 84.1-85.2, compared to the CDC reported national average of 78.6-78.9 (p < 0.001). As shown in Figure 1, KPMAS members overall experienced increased longevity regardless of age. KPMAS members experienced a life expectancy at birth that typically exceeded the national average of other members of their race or gender by 6-10 years (depending on race, sex, and year), with confidence intervals of approximately ± 0.5 years.

**Table 2.** Kaiser Permanente Mid-Atlantic States and Centers for Disease Control and Prevention life expectancy by year and race, with 95% confidence intervals

Year	MAPMG overall life expectancy (lower CI, upper CI)	CDC overall life expectancy	MAPMG Hispanic life expectancy (lower CI, upper CI)	CDC Hispanic life expectancy	MAPMG non-Hispanic Black life expectancy (lower CI, upper CI)	CDC non-Hispanic Black life expectancy	MAPMG non-Hispanic White life expectancy (lower CI, upper CI)	CDC non-Hispanic White life expectancy
2014	84.6 (84.3, 85.0)	78.9	90.0 (88.8, 91.2)	82.1	84.4 (83.8, 85.1)	75.3	85.3 (84.8, 86.0)	78.8
2015	84.7 (84.3, 85.1)	78.7	88.8 (87.6, 90.1)	81.9	85.0 (84.5, 85.7)	75.1	85.0 (84.5, 85.5)	78.7
2016	84.1 (83.7, 84.5)	78.7	89.9 (88.7, 91.1)	81.8	83.7 (83.2, 84.3)	74.9	84.4 (83.9, 84.9)	78.6
2017	85.2 (84.9, 85.6)	78.6	90.7 (89.6, 91.9)	81.8	84.9 (84.5, 85.5)	74.9	85.0 (84.5, 85.5)	78.5

CDC = Centers for Disease Control and Prevention; CI = confidence interval; MAPMG = Mid-Atlantic Permanente Medical Group.

Among members who disclosed their racial/ethnic background, non-Hispanic Black, Hispanic, and non-Hispanic White members of KPMAS experienced significantly longer life expectancies than the CDC national averages and had more equivalent Excess Life Expectancy averages across all race/ethnicities (Figure 2, Table 2, Supplementary Table 3 at www.thepermanentejournal.org/files/2020/20.286supp.pdf). Total life expectancy was higher (5.8-6.5 years) for KPMAS non-Hispanic Whites compared to the CDC non-Hispanic White population (Figure 2). Among KPMAS non-Hispanic Blacks, life expectancy gains ranged between 8.8 and 10.0 years over the CDC dataset, with an average of approximately 9.5 years (Figure 2). These comparisons were significant at the α = 0.05 significance level, with p values < 0.001. The national gap between non-Hispanic White and non-Hispanic Black populations ranged between 3.5 and 3.7 years, with an average of approximately 3.6 years. In contrast, non-Hispanic White KPMAS members had approximately 0.4 years of excess life expectancy over KPMAS non-Hispanic Black members. This was a substantial decrease in the difference between the non-Hispanic White and non-Hispanic Black populations, although the difference between them was still statistically significant (p = 0.047 against the null hypothesis that KPMAS non-Hispanic White population has the same life expectancy as the non-Hispanic Black population).

Hispanic KPMAS members experienced a life expectancy that ranged between 88.8 and 90.7 years. During the study period, the KPMAS Hispanic population had life expectancies of 6.9 to 8.9 years over national averages (Table 2, Supplementary Table 3 at www.thepermanentejournal.org/files/2020/20.286supp.pdf). Life expectancy in the KPMAS Asian and Pacific Islander population ranged from 89.8 to 91.1 years during the study period, similar to the KPMAS Hispanic population; however, there was no CDC comparison group available for this population (Supplementary Material at www.thepermanentejournal.org/files/2020/20.286supp.pdf). Within the KPMAS population, 9.0%-10.2% of members without race/ethnicity reported were designated as nonreporters. The study team utilized 3 methodologies to account for unreported race/ethnicity. All statistics remained significant at reported levels.

Both male and female KPMAS members had elevated life expectancy compared to the CDC national averages (Table 3). Female KPMAS members lived an expected 3.6-5.1 years longer than male KPMAS members in the studied time period (Supplementary Table 4 at www.thepermanentejournal.org/files/2020/20.286supp.pdf).

**Table 3.** Kaiser Permanente Mid-Atlantic States life expectancy by year and sex, with 95% confidence intervals

Year	MAPMG overall life expectancy (lower CI, upper CI)	CDC overall life expectancy	MAPMG female life expectancy (lower CI, upper CI)	CDC female life expectancy	MAPMG male life expectancy (lower CI, upper CI)	CDC male life expectancy
2014	84.6 (84.3, 85.0)	78.9	86.3 (85.9, 86.9)	81.3	82.7 (82.2, 83.2)	76.5
2015	84.7 (84.3, 85.1)	78.7	86.9 (86.5, 87.5)	81.1	82.2 (81.7, 82.7)	76.3
2016	84.1 (83.7, 84.5)	78.7	86.6 (86.1, 87.2)	81.1	81.5 (81.0, 82.0)	76.2
2017	85.2 (84.9, 85.6)	78.6	87.2 (86.8, 87.8)	81.1	83.0 (82.5, 83.5)	76.1

CDC = Centers for Disease Control and Prevention; CI = confidence interval; MAPMG = Mid-Atlantic Permanente Medical Group.

## DISCUSSION

In this study, we examined the life expectancy of KPMAS members (overall, by race/ethnicity, and by sex) as compared to the CDC National Vital Statistics data from 2014 to 2017. Across all categories, we found that KPMAS members were estimated to have longer life expectancy than the national averages reported by the CDC.

Although many complex factors impact life expectancy, including factors that correlate with insurance status, this study offers several key contributions to the literature.^36^^,^^37^ Because insured KPMAS members’ access to health care was roughly uniform due to the presence of health insurance, and disparities by race/ethnicity and sex were reduced as compared to the CDC dataset, our data offers insight into how health insurance may contribute to the differences in life expectancy between different insured communities in the United States. Members of the KPMAS health care delivery system experienced significant increases to life expectancy across all subpopulations, especially in the non-Hispanic Black community, compared with the CDC dataset. This finding offers some indication that the non-Hispanic Black community’s lower life expectancy rates seen in the CDC dataset could be at least partly attributable to insurance status, among other factors. When all members of the study population had equal access to care (as evidenced by health insurance coverage), the non-Hispanic Black community’s life expectancy was more comparable to the non-Hispanic White community (although a 0.4 year, statistically significant difference remained).

In terms of differences in life expectancy by sex, the KPMAS data showed a gap in life expectancy between males and females that is also reflected in the literature and the CDC dataset.^38^ The results by sex indicated that KPMAS male and female members had longer overall life expectancies than the CDC national average. Although the KPMAS dataset showed longer overall life expectancies by sex, the gap between male and female KPMAS patients persisted, with female members living an expected 3.6-5.1 years longer than male members. The gap in the KPMAS dataset was nearly identical to the 4.8- to 5.0-year gap recognized in the CDC dataset. As with the above data, extended life expectancy could be at least partly attributable to insurance coverage status, among other factors.

The study outlined here has several strengths. First, Kaiser Permanente has robust age, sex, and race/ethnicity data available for members (Table 1). Recognizing that 9.0%-10.2% of KPMAS members were missing race/ethnicity data (designated as nonreporters), 3 analytic approaches were used to examine the impact of this missing data (Supplementary Material at www.thepermanentejournal.org/files/2020/20.286supp.pdf). None of these approaches resulted in meaningful differences, indicating that the results shown here are fairly generalizable to the entire KPMAS population. Second, KPMAS has a highly diverse racial/ethnic member population, enabling comparisons to the US population (Table 1). Another strength of this study was the integrity of the data used. While Anderson^33^ and many other demographers have worked with high-level census or survey data, we have employed the use of detailed medical records informing our analysis.

The work presented here has some limitations. First, the CDC dataset includes data from both insured and uninsured populations; the KPMAS dataset only includes insured populations, as all KPMAS members have Kaiser Permanente insurance, be it private or government-backed lines of insurance. Robust life expectancy datasets within an exclusively insured population are not readily available, so this limitation was used as an opportunity to scope research around the potential impact of health insurance on life expectancy. Additionally, as stated above, published literature indicates that insurance is only one contributor to life expectancy—many economic, lifestyle, and social factors also influence life expectancy outcomes.^22^^,^^39^^–^^43^ Furthermore, many of these factors influence insurance status. Thus, members of KPMAS examined in this study were likely to benefit from other socioeconomic and lifestyle advantages that could improve their life expectancy. A comparison of the KPMAS population to the national population, examining education, income, and other relevant factors is the focus of future studies. Second, sample sizes among some subpopulations were extremely small in some older cohorts. In these cases, population means were substituted. This methodology, along with small sample sizes among members ages 95 and over, resulted in estimates for longevity over the age of 95 with a large variance. As these estimates were similar to national averages, we anticipated minimal impact on the overall results. Third, the analysis conducted here was limited to the KPMAS geographic service area (Washington DC, Northern Virginia, and Maryland), which may have impacted results, however state-specific CDC data was not readily available.

This study did not include a correction based on geographic distribution of racial groups, socioeconomic status, or other variables that may correlate with insurance status and life expectancy. In principle, adjustments like inverse probability weighting can correct for differences due to socioeconomic risk factors between groups; however, these models require that we have sufficient data over the entire support space.^44^ Metropolitan areas such as the area covered by KPMAS still experience segregation along racial and ethnic lines, meaning that important covariates are strongly correlated with our variable of interest.^45^ Given that our data was already relatively sparse for certain groups and age ranges, we felt that our data was insufficient to distinguish between socioeconomic and racial factors. Reweighted comparisons with the CDC dataset would likely be even more difficult due to sparse data within the KPMAS set, a lack of correlative features in the CDC dataset, and inconsistent definitions between the two datasets. While we suspect that there are several differences between the underlying populations, including geographic, socioeconomic, and comorbidity distributions, which could contribute to the higher life expectancies enjoyed by KPMAS members, characterizing the extent of these differences was beyond the scope of this study. It would be very useful for future research to explore opportunities to correct for such inconsistencies, either by employing larger datasets with denser support over the explanatory variables or by proposing new, innovative methods to correct for these discrepancies.

Lastly, it is apparent from this data that there are other mechanisms at work beyond insurance status. The KPMAS Hispanic and Asian and Pacific Islander communities had very high life expectancies in comparison to the non-Hispanic White and non-Hispanic Black communities. This is consistent with national trends that have been noted elsewhere, and it is a strong indication that this difference is not solely attributable to differences in health care access and insurance among members of these communities.^46^^,^^47^ One variable of interest that we could not include was the country of origin for individuals in these groups. Unfortunately, this data was too sparse to characterize the extent of this difference from national trends. Further research will be required to understand these differences in the KPMAS-insured population.

Health systems can utilize life expectancy data to identify disparities or gaps in life expectancy that could inform clinical or community benefit activities. The CDC reports that life expectancy declines nationwide over the years studied can be attributed to a few key areas including: the opioid epidemic, suicide, and certain diseases (specifically liver disease).^3^^,^^4^^,^^6^ KPMAS is addressing these specific challenges, within the insured population, through innovative clinical programs. KPMAS has taken a patient-centered approach to opioid prescribing and medication management that has shown early promise in reducing high-dose, brand-name, and combination opioid prescriptions.^48^ KPMAS has created triage and referral pathways for members who present with suicidal ideation (Nancy Weinfield, PhD, personal communication December 16, 2019). Liver disease has been a major focus for several years; KPMAS has a robust hepatitis C screening program, a hepatitis B program, and a hepatocellular carcinoma surveillance program to support patients in managing liver health.^49^^–^^51^

Health systems must also be aware of larger demographic trends in life expectancy among member populations. Although the differences in life expectancy between non-Hispanic White and non-Hispanic Black populations are significantly smaller than the CDC dataset, there is still room for improvement in completely closing the gap. Ensuring that racial/ethnic disparities are reduced remains a focus of KPMAS preventive care and acute care programs.^52^ Similarly, the KPMAS dataset reflects the consistently reported, worldwide finding that females have longer life expectancies than males.^12^ Clinical efforts underway must recognize this disparity, particularly as it informs specific disease processes.^53^^,^^54^

In an environment where more health systems and physician groups are moving toward population health management, either in collaboration with self-funded employers, private health insurers, or through accountable care organizations, understanding population level trends will be critical information for providing equitable and affordable care.^55^^,^^56^ This study of life expectancy in the KPMAS-insured population may aid other health care organizations and physician groups in understanding the role of insurance, and the populations served, with the hopes of improving life expectancy for all.

## CONCLUSION

KPMAS-insured Hispanic, non-Hispanic Black, and non-Hispanic White members had significantly higher life expectancies than the comparison dataset in all years examined and reported. There was a gap in life expectancy between males and females that was seen consistently within the KPMAS and the CDC datasets. But the overall KPMAS male and female patient life expectancy was extended in comparison to data from the CDC.

## Supplementary Information

*Supplementary Material is available at* www.thepermanentejournal.org/files/2020/20.286supp.pdf.

## References

[1] Klenk J, Keil U, Jaensch A, Christiansen MC, Nagel G. Changes in life expectancy 1950-2010: Contributions from age- and disease-specific mortality in selected countries. Popul Health Metr 2016 May;14(1):20. DOI: 10.1186/s12963-016-0089-x 27222639 PMC4877984

[2] Beltrán-Sánchez H, Soneji S, Crimmins EM. Past, present, and future of healthy life expectancy. Cold Spring Harb Perspect Med 2015 Nov;5(11):a025957 DOI: 10.1101/cshperspect.a025957 26525456 PMC4632858

[3] Redfield RR. CDC Director’s media statement on U.S. life expectancy. Accessed December 5, 2019. https://www.cdc.gov/media/releases/2018/s1129-US-life-expectancy.html

[4] Devitt M. CDC data show U.S. life expectancy continues to decline. Accessed December 5, 2019. https://www.aafp.org/news/health-of-the-public/20181210lifeexpectdrop.html

[5] Woolf SH, Chapman DA, Buchanich JM, Bobby KJ, Zimmerman EB, Blackburn SM. Changes in midlife death rates across racial and ethnic groups in the United States: Systematic analysis of vital statistics. BMJ 2018 Aug 15;362:k3096. DOI: 10.1136/bmj.k3096 30111554 PMC6092678

[6] U.S. Department of Health and Human Services. Death rates up for 5 of the 12 leading causes of death. Accessed December 5, 2019 . https://www.cdc.gov/media/releases/2018/p0920-death-rates-up.html

[7] U.S. Department of Health and Human Services; Centers for Disease Control and Prevention; National Center for Health Statistics. Health, United States, 2018. Accessed December 5, 2019. https://stacks.cdc.gov/view/cdc/82212

[8] Olshansky SJ, Antonucci T, Berkman L, et al. Differences in life expectancy due to race and educational differences are widening, and many may not catch up. Health Aff (Millwood) 2012 Aug;31(8):1803–13. DOI: 10.1377/hlthaff.2011.0746 22869659

[9] Cunningham TJ, Croft JB, Liu Y, Lu H, Eke PI, Giles WH. Vital signs: Racial disparities in age-specific mortality among Blacks or African Americans - United States, 1999-2015. MMWR Morb Mortal Wkly Rep 2017 May 5;66(17):444–56. DOI: 10.15585/mmwr.mm6617e1 28472021 PMC5687082

[10] Woolf SH, Schoomaker H. Life expectancy and mortality rates in the United States, 1959-2017. JAMA 2019 Nov;322(20):1996–2016. DOI: 10.1001/jama.2019.16932 31769830 PMC7146991

[11] Kirby JB, Kaneda T. Unhealthy and uninsured: exploring racial differences in health and health insurance coverage using a life table approach. Demography 2010 Nov;47(4):1035–51. DOI: 10.1007/BF03213738 21308569 PMC3000037

[12] Seifarth JE, McGowan CL, Milne KJ. Sex and life expectancy. Gend Med 2012 Dec; 9(6):390–401. DOI: 10.1016/j.genm.2012.10.001 23164528

[13] Bond MJ, Herman AA. Lagging life expectancy for Black men: A public health imperative. Am J Public Health 2016 Jul;106(7):1167–9. DOI: 10.2105/AJPH.2016.303251 27285253 PMC4984780

[14] White K, Haas JS, Williams DR. Elucidating the role of place in health care disparities: The example of racial/ethnic residential segregation. Health Serv Res 2012 Jun; 47(3 Pt 2):1278–99. DOI: 10.1111/j.1475-6773.2012.01410.x 22515933 PMC3417310

[15] Chetty R, Stepner M, Abraham S, et al. The association between income and life expectancy in the United States, 2001-2014. JAMA 2016 Apr;315(16):1750–66. DOI: 10.1001/jama.2016.4226 27063997 PMC4866586

[16] Wong MS, Hoggatt KJ, Steers WN, et al. Racial/ethnic disparities in mortality across the Veterans Health Administration. Health Equity 2019 Apr;3(1):99–108. DOI: 10.1089/heq.2018.0086 31289768 PMC6608703

[17] Stringhini S, Carmeli C, Jokela M, et al. LIFEPATH consortium. Socioeconomic status and the 25 × 25 risk factors as determinants of premature mortality: A multicohort study and meta-analysis of 1·7 million men and women. Lancet 2017 Mar;389(10075):1229–37. DOI: 10.1016/S0140-6736(16)32380-7 28159391 PMC5368415

[18] DuGoff EH, Canudas-Romo V, Buttorff C, Leff B, Anderson GF. Multiple chronic conditions and life expectancy: A life table analysis. Med Care 2014 Aug;52(8):688–94. DOI: 10.1097/MLR.0000000000000166 25023914

[19] Jia H, Lubetkin EI, Barile JP, et al. Quality-adjusted life years (QALY) for 15 chronic conditions and combinations of conditions among US adults aged 65 and older. Med Care 2018 Aug;56(8):740–6. DOI: 10.1097/MLR.0000000000000943 29939910

[20] Sommers BD, Long SK, Baicker K. Changes in mortality after Massachusetts health care reform: A quasi-experimental study. Ann Intern Med 2014 May;160(9):585–93. DOI: 10.7326/M13-2275 24798521

[21] Sohn H. Racial and ethnic disparities in health insurance coverage: Dynamics of gaining and losing coverage over the life-course. Popul Res Policy Rev 2017 Apr;36(2): 181–201. DOI: 10.1007/s11113-016-9416-y 28366968 PMC5370590

[22] Woolhandler S, Himmelstein DU. The relationship of health insurance and mortality: Is lack of insurance deadly? Ann Intern Med 2017 Sep;167(6):424–31. DOI: 10.7326/M17-1403 28655034

[23] National Research Council (US) Panel on Understanding Divergent Trends in Longevity in High-Income Countries. Explaining divergent levels of longevity in high-income countries: The role of health care. Accessed December 5, 2019. https://www.ncbi.nlm.nih.gov/books/NBK62376/# 21977544

[24] Narayan KMV, Patel SA, Cunningham SA, Curran J. Ominous reversal of health gains in the United States: Time to rethink research priorities? Ann Intern Med 2019 Mar; 170(5):330–1. DOI: 10.7326/M18-3653 30743267

[25] Blüher M. Obesity: Global epidemiology and pathogenesis. Nat Rev Endocrinol 2019 May;15(5):288–98. DOI: 10.1038/s41574-019-0176-8 30814686

[26] Bor J, Cohen GH, Galea S. Population health in an era of rising income inequality: USA, 1980-2015. Lancet 2017 Apr;389(10077):1475–90. DOI: 10.1016/S0140-6736(17)30571-8 28402829

[27] Kaplan RM, Milstein A. Contributions of health care to longevity: A review of 4 estimation methods. Ann Fam Med 2019 May;17(3):267–72. DOI: 10.1370/afm.2362 31085531 PMC6827626

[28] Kaiser Permanente Mid-Atlantic States. How care & coverage makes your life better. Accessed December 5, 2019. https://thrive.kaiserpermanente.org/care-near-mid-atlantic

[29] Basra S, Ter-Minassian M, Derus AJ, Watson ES, Horberg MA. All-cause mortality production table from the National Death Index. Presented at: 2020 Health Care Systems Research Network Annual Conference; April 8--10, 2020. Philadelphia, PA. J Patient Cent Res Rev. 2020;7:64--137. doi: 10.17294/2330-0698.1762

[30] Derose SF, Contreras R, Coleman KJ, Koebnick C, Jacobsen SJ. Race and ethnicity data quality and imputation using U.S. Census data in an integrated health system: the Kaiser Permanente Southern California experience. Med Care Res Rev 2013 Jun;70(3):330–45. DOI: 10.1177/1077558712466293 23169896

[31] Elliott MN, Fremont A, Morrison PA, Pantoja P, Lurie N. A new method for estimating race/ethnicity and associated disparities where administrative records lack self-reported race/ethnicity. Health Serv Res 2008 Oct;43(5 Pt 1):1722–36. DOI: 10.1111/j.1475-6773.2008.00854.x 18479410 PMC2653886

[32] Elliott MN, Morrison PA, Fremont A, McCaffrey DF, Pantoja P, Lurie N. Using the Census Bureau’s surname list to improve estimates of race/ethnicity and associated disparities. Health Serv Outcomes Res Methodol 2009 Apr;9(2):69–83. DOI: 10.1007/s10742-009-0047-1

[33] Anderson RN. Method for constructing complete annual U.S. life tables. National Center for Health Statistics. Vital Health Stat 2 (129). 1999 Available at: https://www.cdc.gov/nchs/data/series/sr_02/sr02_129.pdf. Accessed December 5, 2019. 11824050

[34] Efron B. Bootstrap methods: Another look at the jackknife. Ann Stat 1979 Jan;7(1):1–26. DOI: 10.1214/aos/1176344552

[35] Efron B, Tibshirani RJ. Hypothesis testing with the bootstrap. In: An introduction to the bootstrap. Chapman & Hall/CRC: 1993; p 220–36. 10.1201/9780429246593

[36] Institute of Medicine (US) Committee on the Consequences of Uninsurance. Who goes without health insurance? Who is most likely to be uninsured? In: Coverage matters: Insurance and health care. Washington, DC: National Academies Press (US): 2001; p 59--70 25057573

[37] United States Census Bureau. Selected characteristics of the uninsured in the United States. Accessed December 5, 2019. https://data.census.gov/cedsci/table?q=characteristics%20of%20the%20uninsured&tid=ACSST1Y2019.S2702

[38] OECD, Health at a glance 2019: United States. Accessed December 5, 2019. https://www.oecd.org/unitedstates/health-at-a-glance-united-states-EN.pdf

[39] Dickman SL, Himmelstein DU, Woolhandler S. Inequality and the health-care system in the USA. Lancet 2017 Apr;389(10077):1431–41. DOI: 10.1016/S0140-6736(17)30398-7 28402825

[40] Moses H III, Matheson DH, Dorsey ER, George BP, Sadoff D, Yoshimura S. The anatomy of health care in the United States. JAMA 2013 Nov;310(18):1947–63. DOI: 10.1001/jama.2013.281425 24219951

[41] Li Y, Pan A, Wang DD, et al. Impact of healthy lifestyle factors on life expectancies in the US population. Circulation 2018 Jul;138(4):345–55. DOI: 10.1161/CIRCULATIONAHA.117.032047 29712712 PMC6207481

[42] Avendano M, Kawachi I. Why do Americans have shorter life expectancy and worse health than do people in other high-income countries? Annu Rev Public Health 2014 Mar;35(1):307–25. DOI: 10.1146/annurev-publhealth-032013-182411 24422560 PMC4112220

[43] James J. Health Affairs policy brief: The Oregon health insurance experiment. July 16, 2015. Accessed December 5, 2019. https://www.healthaffairs.org/do/10.1377/hpb20150716.236899/full/

[44] Zhou Y, Matsouaka RA, Thomas L. Propensity score weighting under limited overlap and model misspecification. Stat Methods Med Res 2020 Dec;29(12):3721–56. DOI: 10.1177/0962280220940334 32693715

[45] Schuetz J, Larrimore J, Merry EA, Robles BJ, Tranfaglia A, Gonzales A. Are central cities poor and non-white? J Hous Econ 2018 Jun;40:83–94. DOI: 10.1016/j.jhe.2017.11.001

[46] Lariscy JT, Nau C, Firebaugh G, Hummer RA. Hispanic-White differences in lifespan variability in the United States. Demography 2016 Feb;53(1):215–39. DOI: 10.1007/s13524-015-0450-x 26682740 PMC4771518

[47] Acciai F, Noah AJ, Firebaugh G. Pinpointing the sources of the Asian mortality advantage in the USA. J Epidemiol Community Health 2015 Oct;69(10):1006–11. DOI: 10.1136/jech-2015-205623 26034046 PMC4567918

[48] Permanente Medical Staff, A Patient-Centered Approach to Addressing the Opioid Epidemic. Available at: https://mydoctor.kaiserpermanente.org/mas/news/a-patient-centered-approach-to-addressing-the-opioid-epidemic-1759159. Accessed December 5, 2019.

[49] Jonas MC, Loftus B, Horberg MA. The road to hepatitis C virus cure: Practical considerations from a health system’s perspective. Infect Dis Clin North Am 2018 Jun;32(2):481–93. DOI: 10.1016/j.idc.2018.02.007 29778267

[50] Jonas MC, Rodriguez CV, Redd J, Sloane DA, Winston BJ, Loftus BC. Streamlining screening to treatment: The hepatitis C cascade of care at Kaiser Permanente Mid-Atlantic States. Clin Infect Dis 2016 May;62(10):1290–6. DOI: 10.1093/cid/ciw086 26908812

[51] Jonas C. Transforming hepatitis B care in Kaiser Permanente Mid-Atlantic States through a registry and coordinator supported pathway. Oral presentation at: 2019 Health Care Systems Research Network Annual Conference. April 9 2019; Portland, OR

[52] Horberg MA, Hurley LB, Klein DB, et al. The HIV care cascade measured over time and by age, sex, and race in a large national integrated care system. AIDS Patient Care STDS 2015 Nov;29(11):582–90. DOI: 10.1089/apc.2015.0139 26505968

[53] Carrero JJ, Hecking M, Chesnaye NC, Jager KJ. Sex and gender disparities in the epidemiology and outcomes of chronic kidney disease. Nat Rev Nephrol 2018 Mar; 14(3):151–64. DOI: 10.1038/nrneph.2017.181 29355169

[54] Kautzky-Willer A, Harreiter J, Pacini G. Sex and gender differences in risk, pathophysiology and complications of type 2 diabetes mellitus. Endocr Rev 2016 Jun;37(3):278–316. DOI: 10.1210/er.2015-1137 27159875 PMC4890267

[55] Latest ACO Trends Identified. 2018: HFMA. Available at: https://www.hfma.org/topics/news/2018/04/60396.html. Accessed December 5, 2019.

[56] Medicare Shared Savings Program Fast Facts 2018. Available at: https://www.cms.gov/medicare/medicare-fee-for-service-payment/sharedsavingsprogram/downloads/ssp-2018-fast-facts.pdf. Accessed December 5, 2019.

