# Supplementary material for: Life Expectancy Trends Among Integrated Health Care System Enrollees, 2014–2017
Source: Perm J. 2021 Dec 14;25:20.286. doi: 10.7812/TPP/20.286 (PMC8784056; doi:10.7812/TPP/20.286)
Supplement: Supplementary file 1 [file 20.286supp.pdf]

Supplementary Figures

Supplementary Figure 1:

<table>
<tr><th>Race</th><th>2014 Excess Life Plot</th><th>2017 Excess Life Plot</th></tr>
<tr><td>Hispanic</td><td>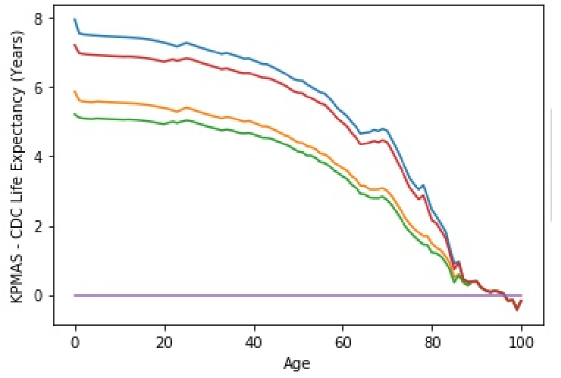
</td><td>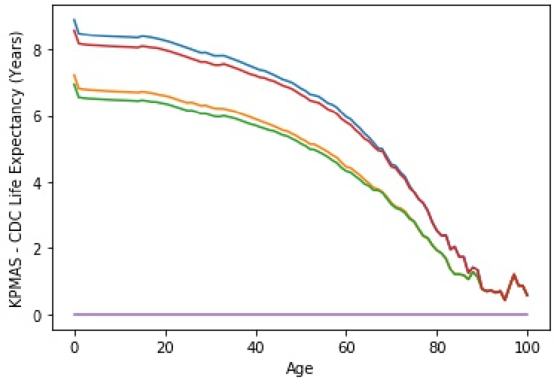
</td></tr>
<tr><td>Non-Hispanic Black</td><td>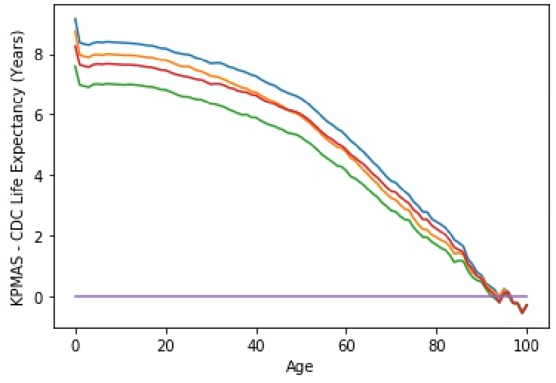
</td><td>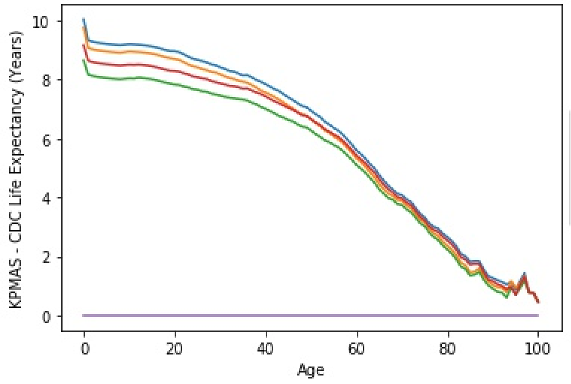
</td></tr>
<tr><td>Non-Hispanic White</td><td>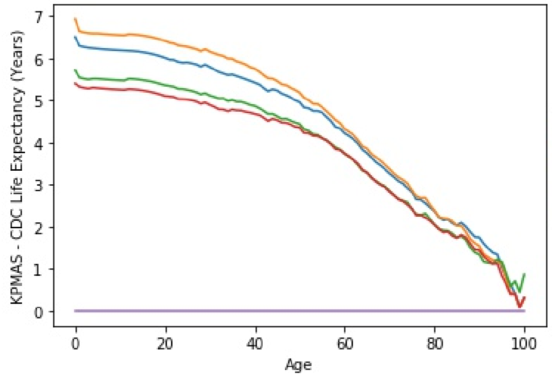
</td><td>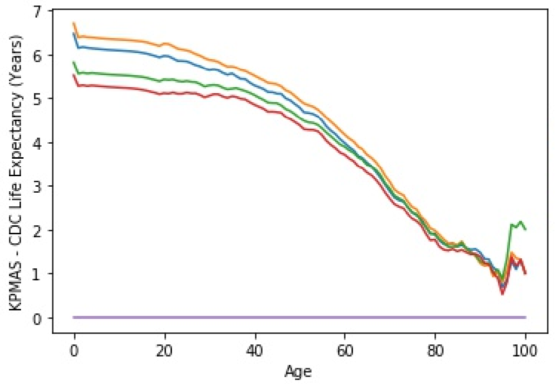
</td></tr>
</table>

Legend

- **Impute None (use only known racial data)**
- **Impute Known (use only members with known racial data, but impute race)**
- **Impute All (use all members and imputes all racial data)**
- **Impute Non-Reporter (uses all members and imputes racial data only for members with Non-Reporter race)**

Supplementary Figure 2: Life Expectancy for Kaiser Permanente Mid-Atlantic States (KPMAS) Asian and Pacific Islander Population, by Year, with 95% Confidence Intervals

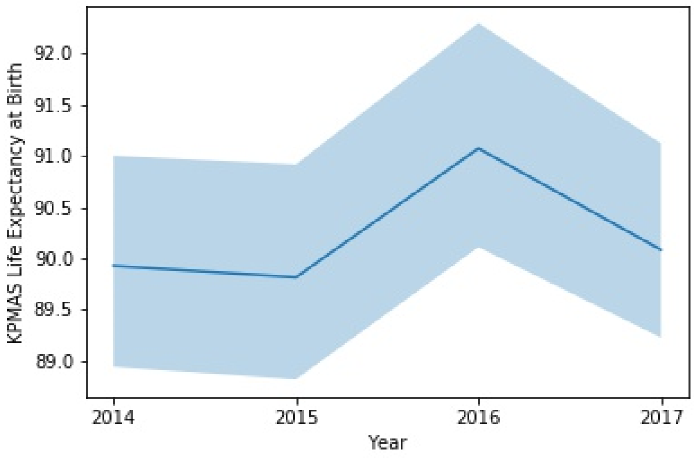

Supplementary Materials:

Non-Reporters

Members who chose not to disclose their race/ethnicity constituted a minority of the dataset (9.0-10.2%; Supplementary Table 1). These patients experienced significantly worse life expectancy outcomes than any group that disclosed racial information, with life expectancies that ranged from 71.2 to 76.2 years. This effect may arise because members who interact with the medical system are significantly more likely to report their race. Supplementary Table 1 presents the life expectancy at birth for non-reporters by year. This life expectancy varied widely, especially in comparison to known populations.

Accounting for Non-Reporters

To examine the impact of non-reported race/ethnicity information on our study, we perform racial imputation using three different methodologies. In the first methodology, we impute only those members for whom we had no recorded racial data, and we combine this information with our known racial data to create a composite life expectancy with the best available information for all members. In addition, we impute race only for patients for whom there is recorded racial data; this allows us to estimate the quality of our imputation and the effect this imputation may have on the Non-Reporter population. Finally, we impute racial categorizations for all members of our population. This final imputation methodology helps us to incorporate information from all members equally.

Supplementary Table 1: Life Expectancy at Birth for Non-Reporters by Year

Year	**Non-Reporter Percent of Population**	**Non-Reporter Percent of Deaths**	**Non-Reporter Life Expectancy**
2014	9.0%	14.0%	71.2 (69.6, 72.9)
2015	10.2%	11.9%	74.3 (72.8, 75.8)
2016	9.5%	12.2%	72.2 (70.8, 73.6)
2017	9.2%	10.5%	76.2 (74.9, 77.8)

Evaluation of Race/Ethnicity Imputation

To evaluate the imputation methodology, we impute data for those patients that have already identified their race/ethnicity. By and large, we find that the GEMS data is relatively good at classifying our members. The method tends to overweight the probability of a member being Non-Hispanic White and to underweight the probability of a member being an Asian or Pacific Islander. To demonstrate this tendency, we present a table for 2015 members showing the average GEMS probability of members coming from each race, grouped by members' reported race.

Supplementary Table 2: Average GEMS Probability of Members, by Race

Self-Reported Race	**Probability Asian and Pacific Islander**	**Probability Hispanic**	**Probability Non-Hispanic Black**	**Probability Non-Hispanic White**	**Probability Other**
Asian and Pacific Islander	62.6%	6.2%	6.5%	21.6%	3.0%
Hispanic	4.5%	67.5%	7.5%	19.3%	1.3%
Non-Hispanic Black	1.8%	3.1%	71.6%	21.6%	1.9%
Non-Hispanic White	3.0%	4.3%	12.1%	78.4%	2.2%
Other	18.2%	12.5%	23.9%	41.9%	3.5%
Non-Reporter	12.8%	16.3%	28.9%	40.0%	2.1%

We can also construct this kind of table for patient deaths. When we do so, we find similar overall tendencies; however, small departures can also lead to significant difference in life expectancy between algorithms.

In addition to examining the raw accuracy of this imputation, we examine how the methodology influences our conclusions. For most races, we find that imputation does not significantly alter our interpretation; however, imputation leads to a significant decrease in the estimated life expectancy of the Hispanic population. This effect arises because of the tendency of the partial allocation imputation method to cause mean-reversion. Because the Hispanic population has a high life expectancy, this effect significantly deflates their estimated life expectancy. The effect is muted in the Asian and Pacific Islander population because members outside of this population are unlikely to be misclassified into it.

This discrepancy indicates that we should treat imputation with a certain level of scrutiny, especially when using it to estimate the life expectancy of the KPMAS Hispanic population.

Effects of Racial Imputation

After performing all three imputation methods, we found that the overall effect of this data was marginal on all populations. We present this series of excess life plots below for completeness; however, the differences between these methods were not statistically significant in most cases.

For Hispanic members, imputation methods which resample the population of known Hispanic members (the green and orange lines) led to large underestimates of life expectation. In addition, inclusion of non-reporters had a slight impact on the population of Non-Hispanic White members (approximately 0.5 to 1 years of life expectation at birth, depending on the imputation methodology and year). This occurs because a significant proportion of non-reporters are estimated to be Non-Hispanic White.

We present the effects of the various imputation methods on our estimates of excess life expectation for the Hispanic, Non-Hispanic White, and Non-Hispanic Black populations below. Overall, we find that inclusion of Non-Reporters through imputation leads to very modest decreases in life expectation (the red and green lines); however, we also see that resampling only our known population (the orange line) can lead to significant problems, especially for the Hispanic population. Because of this instability, we decided to present the known population as our primary estimation methodology.

Despite these complications, conclusions regarding the life expectation of KPMAS members from these racial groups remain largely unaffected by the inclusion or exclusion of non-reporters. Unless we inaccurately resample our known population, inclusion of non-reporters only creates marginal effects that do not alter the primary conclusions of this study.

Supplementary Figure 1

Life Expectancy at Birth, by Year, Asian and Pacific Islander Population

The Asian and Pacific Islander population at KPMAS experienced very high life expectancy during the study period. Life expectancy in this population ranged from 89.8 to 91.1 years during the study period, very similar to our Hispanic population. Unfortunately, the CDC does not publish statistics on the national average life expectancy for Asian and Pacific Islanders, so we cannot compare the KPMAS member population to the national average.

Supplementary Figure 2

Excess Life Expectancy

In Tables 2 and 3, present a detailed look at average life expectancy at birth among members of KPMAS and the CDC. Here we present Supplementary Tables 3 and 4, which demonstrate the Excess Life Expectancy of KPMAS members over the US National Average, grouped by sex, race, and year.

Supplementary Table 3: Kaiser Permanente Mid-Atlantic States (KPMAS) Excess Life Expectancy Over Centers for Disease Control and Prevention (CDC) Dataset, by Year and Race, with 95% Confidence Intervals

Year	**MAPMG Overall Excess Life Expectancy (Lower CI, Upper CI)**	**MAPMG Hispanic Excess Life Expectancy (Lower CI, Upper CI)**	**MAPMG Non-Hispanic Black Excess Life Expectancy (Lower CI, Upper CI)**	**MAPMG Non-Hispanic White Excess Life Expectancy (Lower CI, Upper CI)**
2014	5.7 (5.4,6.1)	7.9 (6.7,9.1)	9.1 (8.5,9.8)	6.5 (6.0,7.2)
2015	6.0 (5.6,6.4)	6.9 (5.7,8.2)	9.9 (9.4,10.6)	6.3 (5.8,6.8)
2016	5.4 (5.0,5.8)	8.1 (6.9,9.3)	8.8 (8.3,9.4)	5.8 (5.3,6.3)
2017	6.6 (6.3,7.0)	8.9 (7.8,10.1)	10.0 (9.6,10.6)	6.5 (6.0,7.0)

Supplementary Table 4: Kaiser Permanente Mid-Atlantic States (KPMAS) Excess Life Expectancy Over Centers for Disease Control and Prevention (CDC) Dataset, by Year and Sex, with 95% Confidence Intervals

Year	**MAPMG Overall Excess Life Expectancy (Lower CI, Upper CI)**	**MAPMG Female Excess Life Expectancy (Lower CI, Upper CI)**	**MAPMG Male Excess Life Expectancy (Lower CI, Upper CI)**
2014	5.7 (5.4,6.1)	5.0 (4.6,5.6)	6.2 (5.7,6.7)
2015	6.0 (5.6,6.4)	5.8 (5.4,6.4)	5.9 (5.4,6.4)
2016	5.4 (5.0,5.8)	5.5 (5.0,6.1)	5.3 (4.8,5.8)
2017	6.6 (6.3,7.0)	6.1 (5.7,6.7)	6.9 (6.4,7.4)
